# A tool to analyze the transferability of health promotion interventions

**DOI:** 10.1186/1471-2458-13-1184

**Published:** 2013-12-16

**Authors:** Linda Cambon, Laetitia Minary, Valery Ridde, François Alla

**Affiliations:** 1 Université de Lorraine, Université Paris Descartes, Apemac, EA 4360, Nancy F-54 000, France; 2 INSERM, CIC-EC, CIE6, Nancy F-54 000, France; 3 CHU Nancy, Epidémiologie et Evaluation Cliniques, Nancy F-54 000, France; 4 Department of Social and Preventive Medicine, Montreal School of Public Health, CRCHUM, 3875 Saint-Urbain, Montreal QC H2W 1 V1, Canada

**Keywords:** Transferability, Health promotion, Intervention, Implementation, Evidence-based health promotion, Knowledge transfer

## Abstract

**Background:**

Health promotion interventions are often complex and not easily transferable from one setting to another. The objective of this article is to present the development of a tool to analyze the transferability of these interventions and to support their development and adaptation to new settings.

**Methods:**

The concept mapping (CM) method was used. CM is helpful for generating a list of ideas associated with a concept and grouping them statistically. Researchers and stakeholders in the health promotion field were mobilized to participate in CM and generated a first list of transferability criteria. Duplicates were eliminated, and the shortened list was returned to the experts, scored for relevance and grouped into categories. Concept maps were created, then the project team selected the definitive map. From the final list of criteria thus structured, a tool to analyze transferability was created. This tool was subsequently tested by 15 project leaders and nine experts.

**Results:**

In all, 18 experts participated in CM. After testing, a tool, named ASTAIRE, contained 23 criteria structured into four categories: population, environment, implementation, and support for transfer. It consists of two tools—one for reporting data from primary interventions and one for analyzing interventions’ transferability and supporting their adaptation to new settings.

**Conclusion:**

The tool is helpful for selecting the intervention to transfer into the setting being considered and for supporting its adaptation. It also facilitates new interventions to be produced with more explicit transferability criteria.

## Background

Health promotion is the process that gives people the means to have more control over their own health and to improve it, from the standpoint of reducing social inequities in health [1,2]. To achieve this requires concerted action on all the social determinants of health, such as early childhood living conditions, schooling, the nature of work and working conditions, the physical characteristics of the built environment, and the quality of the natural environment, etc. This is especially important because, depending on the nature of these environments, the material conditions, psychosocial support, and behavioural patterns are not the same for all groups, rendering different groups more or less vulnerable to health problems.

As such, interventions in this field are often considered complex both to implement and to evaluate [3-6]. The intervention developer or evaluator therefore needs a deep understanding of the theoretical foundations that underlie and explain the intervention and of their capacity to produce an effect or not [7]. From this standpoint, the intervention context becomes a major determinant of the result. This raises the question of the transferability of these interventions, i.e., the extent to which the result of one intervention in a given context can be achieved in another context [8].

This transferability depends upon the conditions of the implementation: e.g. whether or not an experimental protocol was followed by the providers; whether there were incentives in place to encourage and sustain the participation of recipients; whether the providers were trained and supported in implementing the intervention and, where necessary, in adjusting it to the new context. This refers to ‘the dose of the intervention’ [9]. Hence, even when an intervention is replicated exactly, results can vary. Indeed, as described by Victora [9], there can be differences in the relationship between the intervention and the result without differences in the actual dose of the intervention delivered to the target population.

This phenomenon can result from the presence of certain factors in the intervention’s environment, such as antagonistic interventions. It can also be related to recipient-specific factors, such as a past experience creating mistrust or cognitive dissonance [10]. Victora [9] thus identified a certain number of categories of effect modification that could occur without differences in the dose of the intervention delivered.

Factors influencing transferability have been described previously [11]. However, there is as yet no structured tool with which to evaluate, from the stakeholder/provider’s standpoint, the transferability of an intervention. Although tools do exist, they essentially focus on applicability, or on processes for adapting an intervention, or constitute preliminary analyses relating to transferability, yet without being structured [8,11-13] or published as an operational grid for stakeholders [14-16]. The need for the development of a such tool has been emphasized previously [16]. Such a tool could be used to compare settings and, from that, to explore the intervention’s capacity to produce, in the new setting, the same effects as were produced in the first setting. This analysis would be helpful in choosing adaptations to the intervention that would be best suited to the new context and in supporting the transfer and any modifications that might be required. This tool would advance the development of evidence-based health promotion (EBHP) by facilitating the implementation of interventions carried out in other contexts [8,11]. It would thereby also foster a closer connection between research and public health programming of effective interventions.

The objective of this article is to present a tool to analyze transferability and to support the development and adaptation of health promotion interventions to new settings.

In this article, we will refer to the context in which the intervention was produced as the ‘primary’ setting, and that to which it was transferred as the ‘replica’ setting. The interventions themselves will be referred to as the primary intervention and the replica intervention.

## Methods

The tool was created in two stages.

### Developing the tool

We used the concept mapping (CM) method structured according to the six phases proposed by Trochim [17]. This consensus method was designed to enable a panel of experts, through brainstorming, to identify the main components and dimensions of a given reality and how they relate to each other. With this method, qualitative data can be processed using multivariate statistical analyses that combine into categories, and in the form of conceptual maps, the ideas expressed by the participants; weights can be assigned to them, and results can be presented graphically [18]. As such, this collective consensus method is based not only on group facilitation techniques that foster creativity, but also on rigorous statistical analyses that confer credibility on the groupings and the choices made. Concept mapping thus appeared best suited to our objective.

A project team was formed with four researchers (VR, FA, LC, LM). The process was carried out online using Concept System^©^ (version 4.0.1) software.

### Step 1: preparation

#### Selecting experts

The project team selected an expert panel that combined multidisciplinary competencies in health promotion research (public health, epidemiology, health sociology, health psychology, education sciences) with multidisciplinary competencies in health promotion interventions in areas covering a multiplicity of themes and different living environments. The project team identified 43 experts from the health promotion literature and the networks of the project team members. These experts were health promotion researchers and/or practitioners, each bringing valuable contributions, viewpoints and expertise. We limited our selection to French-speaking experts to avoid any confusion tied to language during the process, given the importance of nuances of meaning in this subject matter. The selection was carried out in a stepwise fashion to ensure diversification in areas of expertise.

#### Preparing instructions

The project team prepared the tools needed to set up the method, as well as materials to support the experts in the process and in particular, a text describing the process and a tutorial to help them navigate the software. The project team formulated the question: “What do you consider to be a criterion for transferability of a health promotion intervention?” and provided a definition of transferability [8].

### Step 2: generating the criteria

The experts carried out the brainstorming exercise individually online by responding to the question. They could put forward as many criteria as they wanted, but each criterion had to refer to a single idea. The project team then pared down the resulting list by eliminating duplicates.

### Step 3: structuring the criteria

The experts then assessed the relevance of the criteria on a scale of 1 to 4 (1–not at all relevant; 4–very relevant). Criteria with an average score under 2 were excluded, since we wanted to retain only the most relevant and ensure the tool would be as applicable as possible, as the tool’s length would affect the effectiveness of its utilization.

Finally, the experts each individually grouped the criteria into categories based on what made the most sense to them [17] and named the categories. A criterion could only be in one category.

### Step 4: representing the criteria

A multivariate analysis (multidimensional scaling [19]) was carried out. This enabled us to represent in two dimensions, and within the space of a graph, the correlational distance between the various criteria. The statements most strongly associated with each other were thus located nearer to each other on the graph. Then a hierarchical cluster analysis [19] grouped all the criteria into categories, or clusters. The procedure, using Ward’s algorithm [20], allowed any number of categories to be produced based on the number of times each criterion was placed in the same category by the participants. The final operation consisted of calculating each criterion’s average relevance score.

### Step 5: interpreting the maps

The project team then analyzed the different conceptual maps created by the software. The objective was to reach a consensus on the optimal number of categories to retain and then to settle on one name for each category.

### Step 6: using the maps

The project team produced an initial version of the tool that established a list of transferability criteria structured into categories. Based on the pragmatic objective of their use by the stakeholders, the research team decided to present criteria in temporal sequence (i.e., before selecting the intervention to be transferred, and then during the planning and implementation of that intervention).

To simplify the tool, criteria that were alike—either because they had the same meaning, the same source (e.g. demographic database, evaluation report), or were in the same utilization time frame—were gathered into a single question. The use of questions was motivated by the fact that the tool would be used to compare intervention contexts and to assess how closely they matched in order to decide whether or not to implement an intervention, and the project team felt that organizing the tool as a questionnaire would facilitate this process for the users. Criteria not retained as global criteria were kept as subcriteria of these questions.

All the questions were designed with binary yes–no responses.

### Testing the tool

The objective was to test the tool on the ground by providers who were, in their practices, currently in a situation of transferring interventions created in other contexts.

#### Population

To identify providers who might be contending with an intervention transfer situation, the project team approached two networks:

• The *VIF (Vivons en Forme)* network: Launched in 2004, this network today involves more than 230 municipalities and municipal communities. Its aim is to help families make lasting changes in their lifestyles by developing, with the involvement of local stakeholders, neighbourhood-based interventions [21].

• The network of *Instances régionales d’éducation pour la santé (IREPS)* [Regional health education associations]: These structures develop health promotion programs of varying scope, on every topic and with a wide variety of intervention modalities. [22].

#### Data collection

These two networks suggested 15 sites (10 VIF and five IREPS) involved in intervention transfers. The inclusion criteria were that the interventions had to be in the health promotion field and had to have arisen from a transfer of interventions tried elsewhere.

For each criterion, the providers were asked to evaluate: its measurability; its relevance; and its comprehensibility.

The providers were also asked to evaluate the tool generally, in terms of: its utility; its appropriateness for selecting, adapting, and re-orienting an intervention; its usability; and the factors facilitating or inhibiting its use.

The tool was distributed to the project leaders of the 15 sites. Data was collected by means of semi-structured telephone interviews between the project leaders and the project team; an interview guide was distributed beforehand.

#### Data analysis

The data collected then underwent inductive content analysis. Based on the results of this analysis the tool and its instructions for use were modified to create a second version.

#### Test of the V2 version

This evaluation was carried out with three of the project leaders who had taken part in the first test, along with four researchers who had been involved in the CM process. The V2 version was emailed to these people, who provided open-ended feedback on the tool’s relevance, its comprehensibility, clarity of utilization context description, potential problems in using the tool, and suggestions to improve it. Results of an inductive analysis of the responses allowed us to create the final version of the tool.

*Ethical issues.* This study did not involve individual subjects or identifying health data. Thus, the regulations regarding human research are not applicable [23]. Professionals who participated gave their consent to be named.

## Results

### The transferability criteria

### Experts mobilized

In all, 18 experts gave their consent to participate: six developers of health promotion programs, seven researchers, and five persons who do both. Two experts worked in Canada and 16 in France.

### List of criteria generated

There were 234 criteria generated; these were subsequently reformulated and standardized, bringing the list down to 74. As seen in Additional file 1, these 74 criteria refer specifically to: the characteristics of the population or of the providers; the intervention environment and how receptive it is to the action; and ‘best practices’ in terms of intervention.

**Figure 1:**
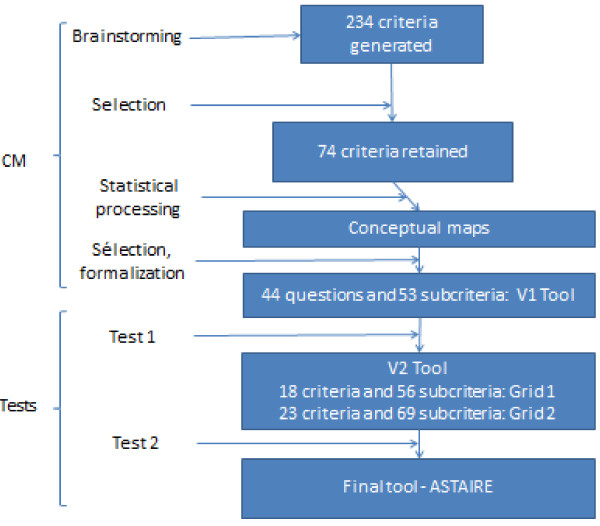
Criteria creation and selection process.

Figure 1 depicts the evolution of the criteria and how they were processed to develop the tool to analyze transferability.

### Conceptual map

The experts’ individual production of categories followed two different logics:

• Source-based, such as data from statistics (health status, age of the population, etc.) or data collected from professionals (existence of antagonistic or synergistic interventions in the intervention setting, mobilization of partnerships, etc.);

• Chronological, such as data collected before the intervention (population characteristics, environmental feasibility conditions, etc.), during the intervention (involvement of stakeholders, accessibility of the intervention, adherence to the projected intervention protocol, etc.), or even after the intervention (such as participation criteria).

**Figure 2:**
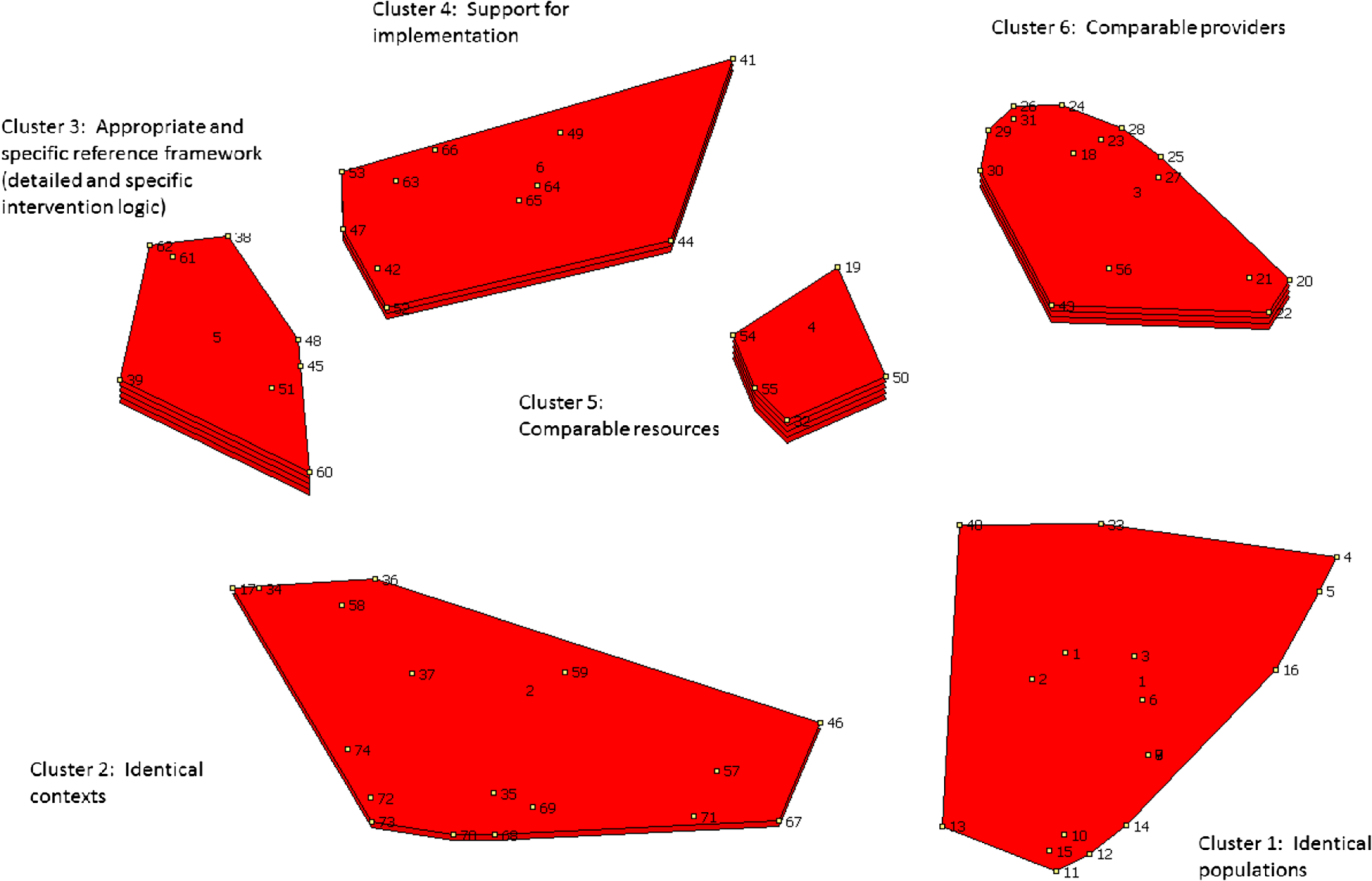
Concept mapping and the transferability of health promotion interventions.

From this foundation, the software generated several conceptual maps. The project team selected the conceptual map with six categories (Figure 2). Collectively, it appeared to be the most relevant because it was in line with the categories of data that were sought or mobilized in planning an intervention.

Each category’s designation was thus defined by the project team (Table 1).

**Table 1:** Designated categories and average scores

**Category**	**Name**	**Content**	**Average score**
Category 1	Comparable populations	Comparability of the characteristics of the primary and replica populations; demand; need	2.86
Category 2	Comparable contexts	Conditions; feasibility; partnerships; adaptability	3.03
Category 3	Appropriate and specific reference framework	Primary intervention characteristics; methodological tools; adaptation	3.2
Category 4	Implementation support	Support for transfer; formal process to assist the transfer	3.34
Category 5	Comparable resources	Financial, human, and material resources	3.33
Category 6	Comparable providers	Support; providers’ skills and capacities; resources	3.13

The project team also refine the list and categorization of criteria:

• Two criteria (19 and 50) were removed because they were represented more clearly elsewhere, and two others because they were redundant (32 redundant with 71, and 40a redundant with 21 and 22).

• Only one criterion was removed because its relevance score was below 2 (67: ‘Changes in other sectors than health have already been supported in this population and were successful’).

• Criterion 10 (‘The health status of the replica population is comparable to that of the original population’) was moved from category 3 (related to appropriate and specific reference framework) to category 1 (related to the population).

It can be seen in Figure 2 that the category related to resources is considered the most relevant. In this figure, categories (clusters) are shown as red fields encompassing criteria, each represented by a point and a number; the categories’ thickness corresponds to the mean of their relevance scores, the thicker ones being considered by the experts to be particularly influential in terms of transferability. The categories related to the providers and to the reference framework are also important. Next in line are implementation support, context, and finally, population characteristics.

The corresponding list of numbered and structured criteria is presented in Additional file 1.

#### Development of the tool

Based on this map-making, we were able to develop the tool. The project team organized it by grouping neighbouring criteria into a single question. Thus, the list went from 74 criteria to 44 questions having 53 subcriteria, all classified under the six categories of the selected conceptual map.

This first version of the tool was then formatted so that it could be tested.

### Result of the testing

The test was carried out by 15 project leaders (one of whom later withdrew from the testing because of time commitments).

#### Quantitative data

All the criteria and subcriteria were found to be relevant, comprehensible, and measurable by the majority of the testers: all the criteria and subcriteria were judged relevant by 10 of 14 testers, to be comprehensible by seven out of 14 testers (except for one that was judged to be comprehensible by only six testers out of 14), and measurable by nine out of 14 testers. As well, one criterion was considered to be unobservable by five testers (‘The socioeconomic characteristics of the primary intervention’s population and those of the replica are the same’).

#### Qualitative data

While the tool’s relevance was not questioned, and it even appeared to be eagerly expected, the project leaders drew attention particularly to the following points:

• The wording of the criteria was overly complex, and this was exacerbated by the question format.

• Certain criteria were not relevant because they were redundant or interrelated with other criteria, or could only be observed at the very end of an intervention and therefore were not useful except in explaining the outcome, or were related more to best practices of intervention than to transferability as such.

• The length of the tool inhibited its use.

• A version was needed for stakeholders or researchers producing a primary intervention that would help in designing and describing it in such a way that it could be transferable.

• The tool needed explanation: when to use it, a definition of transferability, a decision support tool based on the presence or absence of criteria.

• A scoring system was needed that would support decision-making based on meeting the criteria.

• The scoring needed to be defined in a way that did not incorporate judgments.

#### Adjustments

Thus, adjustments were made to both the content and the form of the tool, by removing duplicates and redundancies, rewording criteria to make them more precise (wording each idea concisely using terminology familiar to all types of stakeholders, and replacing the question format with a checklist), and finally, grouping criteria together, as had been done before, i.e., bringing related criteria together under a single, more comprehensive criterion. Criteria grouped in this way then became subcriteria. In doing this, it was essential to ensure that attaching several subcriteria to a single criterion was consistent with the groupings created in the CM step. A few exceptions were made in cases where certain criteria fell into two neighbouring categories and addressed the same main overall idea.

Then the project team restructured the tool into four broad categories of transferability, in line with the structure used in the concept mapping: population, environment, implementation, and support for transfer. An introduction was added that included a glossary and answered the questions: What is transferability? How was the analysis tool designed? How should the analysis tool be used? When should transferability be analyzed?

Finally, in response to the testers’ request for a tool to be used in designing and describing primary interventions, we decided to divide the examination of transferability into three time frames: 1) before the primary intervention is implemented; 2) when selecting and implementing an intervention that has already been tried elsewhere; and 3) when evaluating an intervention that was transferred in this way.

These adjustments resulted in a revised version of the tool, V2, organized into two tools:

Tool 1, to be used in designing and describing a primary intervention, consists of 18 criteria and 56 subcriteria and is used from the start, when the intervention is being conceived, with an emphasis on reporting.

Tool 2, with 23 criteria and 69 subcriteria, is intended to be used when a primary intervention is being considered for transfer to a different context, or when assessing *a posteriori* what caused any difference in effects between the primary intervention and the replica intervention ultimately implemented.

The 18 criteria of the first tool are included in the 23 criteria of the second; the latter is more comprehensive because it includes aspects related to transfer, which are not relevant in the former.

#### Testing V2

This second version was tested by four researchers and three providers.

The tool was considered by most of the experts to be comprehensible (6/7), relevant (6/7) and specific to its utilization context (6/7). Four of the seven experts reported they could encounter difficulties working with this tool; mainly, they felt more support was still needed in interpreting the tool. Overall, the tool was perceived as being quick to use and focused on the most relevant criteria. The tool intended for providers was especially seen as helpful in targeting the adjustments needed for successful implementation.

Suggestions were also made to give the tool a name, modify some of the wordings, deal with the matter of training the providers, make the instructions clearer, and explain the next steps in terms of planning, after the tool has been filled in.

After this last testing, adjustments were made, and a final tool was produced that contained the two tools. This tool is presented in Additional file 2. It was given the name ASTAIRE (for “AnalySe de la Transférabilité et accompagnement à l’Adaptation des Interventions en pRomotion de la santE” – assessment of transferability and adaptation of health promotion interventions).

## Discussion

The objective of this research was to develop and validate a tool to analyze the transferability of health promotion interventions. The premise of this work was that a tool was needed that would, by making it possible to compare settings, be of assistance in choosing the primary intervention most suited to the replica setting and, if necessary, support the process of adapting it to that setting. The present tool can be used to support both stakeholders in transferring interventions, and researchers in considering what parameters could be useful to improve their intervention’s transferability beforehand.

### Criteria that extend beyond the literature

The criteria defined in the tool repeat, to a certain extent, those found in the literature, but with greater detail, formalization, and precision [11]. Indeed, a review of the health promotion literature identified approximately 30 factors related to transferability in the areas of population characteristics, environment, professionals, healthcare system, and method of intervention. These factors were derived from empirical reflections, transfer and adaptation processes, and process evaluations [11]. Some authors have attempted to list these criteria [16], while others have examined external validity criteria [12,15,24,25] or the adaptation process [14]. However, few of the criteria emerging from the literature are structured operationally in a tool that can easily be used by stakeholders wanting to transfer an intervention. Moreover, of the tools that have been produced [14-16], two focus on applicability [14,15,24,25], and only one actually deals with transferability in a distinct manner, albeit marginally, since only six out of 21 questions relate to transferability, and they concern only three dimensions: magnitude of health issue in local setting; magnitude of the “reach” and cost-effectiveness of the intervention; and target population characteristics [16]. These three dimensions can be found in category 1 in ASTAIRE. However, there is consensus that in general most of the criteria are scarcely reported in the literature.

Thus, looking at the categories of ASTAIRE, we see consistencies with the published data [11]. Under the heading ‘Population’ in ASTAIRE, eight criteria (criteria 1–5, 8, 9, and 11) were already found in the literature, and three others (6, 7, and 10) were either newly created or resulted from making more specific or breaking down criteria that were in the literature. In the ‘Environment’ category, two criteria (12 and 13) were found in the literature, and criterion 14 was newly created. In the ‘Implementation’ category, five criteria (15–18 and 20) were in the literature and only criterion 19 was created in ASTAIRE. Finally, in the ‘Support for transfer’ category, criterion 21 was identified in the literature but was defined in greater detail in ASTAIRE with subcriteria. Criteria 22 and 23 were newly created. Thus, of the 23 criteria in ASTAIRE, 16 had been identified in the literature but were made more detailed, worded more precisely, and accompanied by descriptive subcriteria to make them more measurable in ASTAIRE. Seven criteria were new. Distributed over the four categories, these latter criteria added concepts that complemented those in the literature (perception of the intervention’s utility, mobilization of partners, the primary intervention’s suitability for providing contextual elements, the intervention’s acceptability to those implementing it, etc.) and that reflected experience on the ground. As such, this work done with experts contributed real added value by making this list of criteria more complete, specific, and pragmatic.

In addition, we note that all the criteria operate on one or the other, or both, of the levels described by

Victora et al [9], i.e. with and without differences in the actual dose of the intervention delivered to the target population:

• Factors that are intrinsic to the recipient and that reduce the effect of the intervention (criteria 1 to 11).

• Factors that increase the effect of the intervention (*synergism*) (criterion 12).

• The recipient’s real need with regard to the intervention (*curvilinear dose–response association)* (criterion 6).

• The presence or absence of interventions that are antagonistic to the intervention studied (criterion 12).

• The absence of a necessary cofactor in the causal chain of the intervention’s effect (criteria 12 to 14).

• The presence or absence of influential causes that are consonant with the intervention but external to it (criteria 12 to 20).

The three first categories are specific to recipients and the next three, to the environments in which they live.

The commonality of results from both the literature [11,16] and the concept mapping exercise add to the credibility of the tool developed. This tool has, moreover, been enhanced by the knowledge of experts, including both researchers and program developers.

### How providers can use ASTAIRE

To support its use by providers, we structured the tool to be used on two levels. The first is when choosing and implementing, in a new setting (the replica setting), a primary intervention from another setting. This analysis may lead to three conclusions: not to implement the intervention, to implement it with modifications, or to implement it without modifications. The second level occurs when evaluating the replica intervention, i.e., when an *a posteriori* evaluation of the presence or absence of transferability criteria can help to explain the effects of the replica intervention with reference to the effects of the primary intervention. ASTAIRE can thus be incorporated into providers’ practice, since it can be used at different points in project planning.

### How researchers can use ASTAIRE

Because issues related to transferability arise not only during the action of transferring, but well upstream, when the primary intervention is being conceived, it became clear that the tool needed to be structured accordingly. Taking this into account meant rethinking both the methods for developing and evaluating interventions, so that they take into account all the factors that influence results [11], and the models for reporting data. This tool is intended to address the latter aspect. In fact, the issue of reporting is related to the issue of external validity, which is the extent to which the conclusions of one study can be extrapolated to other populations, other contexts, and other times [26]. It is this validity that enables conclusions to be drawn, from the researcher’s standpoint, regarding the intervention’s potential for generalization. To increase this validity, there are tools, such as RE-AIM, intended specifically for use in health promotion studies. While that type of tool can help researchers examine the suitability of their studies for generalization, particularly in relation to the methods used, our tool is intended as a supplement. In effect, it can be used before a new primary intervention is carried out, to identify all the parameters that could influence the effects, and then to carry them forward into the final documents describing the intervention. It thus bridges the issues of external validity and of transferability, by inviting the producers of data, very often researchers, to produce from the moment of an intervention’s conception the specific descriptive elements that can be used to compare contexts. As such, it supplements other tools such as RE-AIM and makes them more specific, and fits with the concept of evaluability assessment, which is used to carry out pre-evaluations or exploratory evaluations of programs in order to optimize the chances of benefiting from useful formal evaluations. The tool fits particularly well with one of the objectives assigned to this concept, which is to promote the transfer of research into practice by examining the feasibility, acceptability, and adaptation of evidence-based practices in new setting or populations [27].

### Strengths, limitations, and perspectives

To develop this tool, we used a structured and validated method, concept mapping. However, even though the project leaders validated the tool based on their own perceptions of the relevance of the criteria and in terms of its practicality, comprehensibility, and ease of use, the impact of each criterion on the results remains to be validated, i.e., the weight of their influence and the mechanisms at work in this influence [25] and a possible synergy of action between these criteria. This is the subject of a prospective ongoing project.

Furthermore, although the initial work was based on a review of international literature, it has only been tested within a French-speaking context. We thus need to ensure that the tool can be adapted to interventions implemented in international contexts. For example, some criteria deemed especially important in France might be less so elsewhere, and vice-versa, thereby modifying the relevance of certain criteria. This is also the subject of the above-mentioned project.

Lastly, it is important to point out that the use of this tool in itself cannot alone change practices. Indeed, to be truly useful in the development of an evidence-based approach, it must fit into a logic-based practice that takes into consideration health promotion practitioners as well as researchers. The premise is that the combination of these two approaches needs to be followed up: how can we make better use of research data in practice or in political decision-making; and how can we better integrate the needs of stakeholders into research orientations? This is the challenge of evidence-informed decision-making (EIDM) [26], defined as “the process of distilling and disseminating the best available evidence from research, practice and experience and using that evidence to inform and improve public health policy and practice.” In the same way, our project fits within the fifth step of evidence-informed public health (EIPH), consisting of adapting research evidence to a local context [27]. This step helps to answer the question: “Can I use this research with my client, community or population?” [12].

These researcher/stakeholder interaction processes are incorporated into policy-making processes, particularly using knowledge transfer methods that coordinate initiatives tied to research data production, dissemination, and use [28].

## Conclusion

In providing support to decisions regarding implementing a specific intervention in a setting, and supporting its adaption if necessary, this tool can help to further the development of the EBHP approach.

This tool can be used both upstream and during the course of implementation for greater transferability. Collecting the data needed to complete the tool could be done during the diagnostic phase in health promotion planning. The tool thereby contributes to developing evidence-based health promotion without compromising the foundation of its intervention logic, which is integration into the context.

Finally, the fact that this tool was structured for use by two potential users—providers and researchers—invites some reflection on the links that exist between these two worlds, which are the worlds of research and of intervention on the ground. Thus, the issue of transferability raised by the this tool’s development brings us back to the issue of a broader knowledge transfer policy [29] or EIDM that could make research more useful and usable by stakeholders, provide stakeholders with better tools for analyzing and transferring data produced by research, and perhaps even get stakeholders more involved in research, and researchers more involved in what is happening on the ground [30-32].

## Abbreviations

CM: Concept Mapping; ASTAIRE [29]: AnalySe de la Transférabilité et accompagnement à l’Adaptation des Interventions en pRomotion de la santE” - -assessment of transferability and adaptation of health promotion interventions; EBPH: Evidence-based health promotion.

## Competing interest

The authors declare they have no conflicts of interest and that the funding sources had no input into the study design, data collection, analysis and interpretation of data, the writing of the report or the decision to submit the paper for publication.

## Authors’ contributions

LC, LM, VR and FA conceived the study, analyzed and interpreted the data, and drafted the paper. All authors read and approved the final manuscript.

## Pre-publication history

The pre-publication history for this paper can be accessed here:

http://www.biomedcentral.com/1471-2458/13/1184/prepub

## Supplementary Material

Additional file 1 Transferability criteria by category. Click here for file

Additional file 2 ASTAIRE. Click here for file
