# Supplementary material for: A tool to analyze the transferability of health promotion interventions
Source: BMC Public Health. 2013 Dec 16;13:1184. doi: 10.1186/1471-2458-13-1184 (PMC3878633; doi:10.1186/1471-2458-13-1184)
Supplement: Additional file 1 — Transferability criteria by category. [file 1471-2458-13-1184-S1.docx]

**Supplementary Data 1: Transferability criteria by category**

**Category 1: Comparable populations**

There is the same relationship of trust between providers and the community where the intervention will be implemented as there was in the primary intervention. 33The population of the replica intervention has the same resources as those of the primary intervention (age, education level, literacy). 6

The replica intervention has the same sociocultural accessibility as did the primary intervention. 3

The population of the replica intervention has the same affective-emotional characteristics (sex, ethnicity, religion, socioeconomic level) as did the population of the primary intervention. 9

The health status of the replica population is comparable to that of the primary population. 10

The population of the replica population has the same perception of its needs / representations of health or of the problem being treated as did the primary population. 11

The replica population has the same perception of its vulnerability as did the primary population. 12

The replica intervention has the same financial accessibility as the primary intervention had, from the recipients’ perspective. 1

The replica intervention has the same geographic accessibility as did the primary intervention. 2

The population of the replica intervention has the same (age-related) cognitive characteristics as did the population of the primary intervention. 7

The population of the replica intervention has the same cultural characteristics (lifestyle and representations of the world) as did the population of the primary intervention. 8

The replica intervention’s participants/recipients are just as motivated / pleased as were those of the primary intervention. 16

Rates of participation are the same in both the replica and the primary interventions. 4

The populations’ degree (qualitative) of participation is identical in the replica and the primary interventions. 5

The recipients believe in the effectiveness, the added value, and the merits of the intervention to be transferred. 13

The replica population expects just as much from the intervention as the primary population did. 14

The levels of incentives (financial, volunteerism, profit-sharing, obligation) are the same in both the primary and replica interventions. 40

**Category 2: Comparable contexts**

The problem this intervention is supposed to resolve is considered a priority by the providers. 17

The participants in the replica intervention believe in its values and objectives just as much as the participants in the primary intervention did. 15

The types of partnerships and other networks that supported the primary intervention and those that can be mobilized for the replica intervention have been identified: community groups, associations, local decision-makers, funding organizations, public agencies. 34

The responsibilities have been divided between the action and decisional levels in the same was as was done in the primary intervention. 44

Changes in other sectors than health have already been carried out successfully in this population. 67

The replica protocol addresses the same population as did the primary protocol. 46

The primary intervention is feasible in this context (technical and political feasibility). 59

The intervention to be transferred satisfies the priorities and needs of the recipients of the replica intervention. 57

The implementation of the replica intervention is aligned with the political and social agendas (pre-existing and durable political will, institutional support affirmed and announced by decision-makers). 72

There are, in the environment and in the characteristics of the replica intervention’s population, all the elements needed to produce the result seen in the primary intervention. 71

In the context of the replica intervention there are past experiences that are not unsupportive / antagonistic to the intervention, or at least no more than existed in the primary intervention. 68

There are no antagonistic actions in the new context, or at least no more than there were in the primary intervention. 69

The work environment / conditions is (are) comparable to that (those) of the primary intervention. 35

A feasibility (pilot) study can be done in the replica context in order to make adaptations. 37

The decision-makers believe in the effectiveness, the added value, and the merits of the intervention to be transferred. 74

The political and social environments are stable during the intervention. 73

There are synergistic actions in the new context, or at least as much as were in the primary intervention. 70

The primary intervention can be adapted to the epidemiologic and sociocultural context of the replica intervention (there is room to manoeuvre in the protocol to be able to adapt to expressed needs and demands). 58

A preliminary diagnosis of the primary intervention is carried out before it is implemented in the new context (meetings of local decision-makers and of population representatives…) and approved by the stakeholders to ensure that the adaptations needed will be possible but will be kept to a minimum. 36

**Category 3: Appropriate and specific reference framework**

The primary intervention is based on common legal structures and regulations and on front-line providers (e.g. teachers, health and social service professionals, etc.) or, if not, plans are in place to make it routine. 60

Flexible methods and tools have been created in order to build upon existing projects and integrate into existing contexts rather than applying a new project implementation model. 38

The replica intervention is built upon a theoretical framework (logical framework, Plan/Do/Check/Act,…), that has specific adjustment mechanisms to allow continuous adaptation to the context (adapting the context to the intervention or the intervention to the context). 39

Modifications have been made to the primary intervention in order to implement it in the replica context. 45

The protocol of the replica intervention uses the same intervention modalities as were used in the primary intervention’s protocol. 48

The strategies for adapting the primary intervention are developed with the different stakeholders, who support them. 51

**Category 4. Support for intervention**

The quality of the management of the replica intervention is high. 41

The tasks assigned to the providers are formalized and have been explained to them in detail. 49

The protocol and/or implementation of the replica intervention takes into account (seeks out) the key lessons to be learned from the primary intervention (failures, weaknesses, or potential counterproductive effects) 47

There is a protocol (formalized procedures, chart, reference framework) that provides a detailed description of the implementation of the primary intervention so that it can be transferred. 61

The tools used by the primary intervention are fully accessible. 64

An evaluation (context, process, results) of the primary initiative has been done to draw lessons in terms of errors to avoid (failures, weaknesses, potential counterproductive effects), key success factors, and the extent of their influence on the results of the intervention; convergent and divergent stages of the primary intervention process were identified. 62

The evaluation of the primary intervention is fully accessible. 63

The results of the replica intervention are measurable and measured regularly to be able to continuously adapt to the context. 53

There is scientific support for the transfer of the intervention, as well as a rigorous evaluation of its process and results. 66

There is an action plan for the replica intervention that is similar to that of the primary intervention. 42

The scale and duration of the intervention are sufficient, in reference to the primary intervention. 52

Links have been established with the teams that did the studies showing the effectiveness of the primary intervention. 65

**Category 5: Comparable resources**

The providers in the replica intervention have set up means of adapting the primary intervention to their context (meetings of local decision-makers and of population representatives, internal competencies, methods of support, etc.….) 19

The noteworthy determining factors (causal chains) of the result in the primary intervention are present in the replica intervention. 32

The objectives of the intervention are clearly defined in collaboration with the local stakeholders and approved by them. 50

The material resources (suitable physical environment, teaching materials), are equivalent to those that were available for the primary intervention. 54

The financial resources are equivalent to those that were available for the primary intervention. 55

**Category 6: Comparable providers**

Providers in the replica intervention have been specifically trained in the primary intervention. 23

The skills of the providers are just as suited to the replica intervention as were those of the providers in the primary intervention. 28

The quality of stakeholder leadership in the replica intervention is as strong as in the primary intervention. 43

The providers believe in the effectiveness, the added value, and the merits of the intervention to be transferred. 18

The project leader has the appropriate expertise, defined in the primary intervention, to implement and adapt the intervention (characteristics, level of competencies, and ability to sustain their use throughout the intervention) 24

The providers have the appropriate expertise, defined in the primary intervention, to implement and adapt the intervention (characteristics, level of competencies, and ability to sustain their use throughout the intervention) 25

The providers of the replica intervention are just as involved in the action as were those of the primary intervention. 20

The providers of the replica intervention have the same financial, scientific and/or professional interest in implementing this action as did the those of the primary intervention. 21

The providers have the same knowledge (scientific / practical-experiential / know-how) of their population. 26

The providers of the replica intervention have the same capacity for team work as did those of the primary intervention. 27

The methods used to train the providers enable them to accept and take ownership of the new intervention. 29

The methods used to support the providers enable them to accept and take ownership of the new intervention. 30

The providers in the replica intervention who do not have the required skills can receive training that will give them the competencies to implement the primary intervention. 31

The human resources (skills, available work time) are equivalent to those that were available in the primary intervention. 56
