# Supplementary material for: A tool to analyze the transferability of health promotion interventions
Source: BMC Public Health. 2013 Dec 16;13:1184. doi: 10.1186/1471-2458-13-1184 (PMC3878633; doi:10.1186/1471-2458-13-1184)
Supplement: Additional file 2 — ASTAIRE. [file 1471-2458-13-1184-S2.docx]

**Supplementary Data 2 – ASTAIRE**

**ASTAIRE^[[1]](#footnote-1)^ :**

**Assessment of transferability and adaptation of health promotion interventions**

**V1–2012**

**User Guide**

**What is transferability?**

Because of their complexity, the effects of health promotion interventions are the product not only of the interventions themselves, but also of the contexts in which they unfold. In this field, even if an intervention has demonstrated its effectiveness in a given setting, the observed effects are rarely identical in another setting; the intervention itself may be applicable, but it may generate other effects than were produced in the primary intervention. Indeed, many characteristics can influence those effects: adjustments in the intervention’s implementation, or characteristics of the population, the healthcare system, the environment, the implementation conditions, etc.

The transferability of an intervention is thus understood in this document to be **the extent to which the effects of an intervention in a given setting can be observed in another setting^[[2]](#footnote-2)^**. It is different from applicability (see Glossary). This question is crucial when we want to transfer an intervention that is complex—as are health promotion or health education interventions—from one context to another and ensure *a priori* that the effects are the same.

**When should transferability be analyzed?**

Transferability can be explored in three different cases:

1. **Before implementing a new intervention** – In this document, we call this type of new intervention ‘primary’. Looking at its potential transferability at this time makes it possible, from the intervention’s conception, to take into account all the parameters that could influence the effects and to carry them forward into the final documents describing the intervention (reports, articles…). All the parameters should be well described in order to facilitate transfer of the intervention by others.
2. **When choosing and implementing an intervention that has been tried elsewhere (the primary intervention) in a new setting** – In this document, we call this type of intervention a ‘replica’. Examining transferability at this point allows us to study the comparability of the setting and, consequently, the intervention’s capacity to produce, in the new setting, the same effects that were seen in the primary intervention. This analysis can lead to one of three decisions: not to implement the intervention, to implement it with modifications, or to implement it without modifications. While the term ‘intervention’ is used generically, it encompasses a variety of situations— programs, community outreach activities, intervention methods (focus groups, workshops, etc.)—that providers may encounter.
3. **When evaluation a ‘replica’ intervention**: An *a posteriori* evaluation of the presence or absence of transferability criteria may help to explain the effects of the ‘replica’ intervention, with reference to the effects of the primary intervention.

**How was the analysis tool designed?**

This tool for analyzing transferability and supporting the adaptation of health promotion interventions was created using a structured method to consult with health promotion experts, researchers, and stakeholders. Then it was subjected, in France, to a progressive validation process, tested in the field by leaders of health promotion projects. Using this method, a certain number of **criteria** were selected, which are presented in this tool. These criteria describe the types of factors that can influence transferability. They are organized into **four broad categories**:

- population: criteria that describe the population
- environment: environmental factors that can influence the effects;
- implementation: elements of the intervention’s implementation, especially with respect to aspects of planning and partnerships;
- support for transfer: elements that support an intervention’s transfer and are especially useful in adapting it to the new setting.

Most of the criteria have been broken down into **one or more subcriteria** which describe the criteria and are thereby helpful in determining whether the criteria are present. Together they form a tool. Responses are indicated by **ticking the checkbox** if that criterion is present.

**How should the analysis tool be used?**

This tool is not intended to replace the usual planning processes used by stakeholders, but rather to be incorporated into them. Basically, it adds elements that are used to analyze the parameters that can influence an intervention’s transferability. Therefore, it is advisable to use it within the following approach:

- A project logic whose structure extends all the way to evaluation: diagnosis, definition of objectives, programming, evaluation.
- Partners involved from the outset, in the design stage, including the population.
- Ethical health promotion principles

This tool has been structured as two tools:

- **A tool to use when designing a primary intervention and carrying forward its descriptive elements** (1^st^ case mentioned above). In this case, the tool can be helpful in order to construct the primary intervention in such a way as to make it transferable, and to describe it in detail for any other stakeholders who may wish to transfer it later. In fact, transferability should be taken into account from the moment the primary intervention is conceived, in parallel with effectiveness evaluation. **This is Tool 1 in this document, which consists of 18 criteria**.
- This tool is to be used at the outset, when the intervention is being designed, as well as when it is being reported on and evaluated.
- **A tool to use when considering the transfer of a primary intervention that has been tried elsewhere into one’s own practice setting** (2^nd^ case mentioned above), or **when assessing *a posteriori* what might have caused any difference in the effects** between the primary intervention and the replica intervention ultimately implemented (3^rd^ case mentioned above). In this case, the tool is used to make a decision regarding implementing the intervention in the replica setting, to adapt it, and to understand the effects of the replica intervention. **This is Tool 2, which consists of 23 criteria.**
- This tool is to be used at several stages of planning:
  - at the diagnostic level, to collect and assess the descriptive characteristics of the population (category 1), the environment (category 2) and of items related to feasibility of implementation (category 3);
  - at the levels of defining objectives, programming, and implementation, to collect and assess the elements characterizing the implementation (category 3);
  - on an ongoing basis, throughout the project cycle, for items related to methods used to support the transfer (category 4);
  - at the time of evaluation, for all four categories.

The use of the tool should thus be part of the project development process. The time needed for its use should be taken into account in planning, at each of the above-mentioned stages.

Once the tool has been completed, the assessment of transferability is not automatic, i.e., the approach is not quantitatively linked to the number of boxes checked. As in every transfer process, when making a judgment regarding whether or not to proceed, the stakeholders using the tool will be guided by their own expertise. This can be done on the basis of the following principles: the weight assigned to each category of criteria, or even to the criteria themselves; the stakeholders’ capacity for adapting the intervention in certain respects to be more in line with the primary intervention, or modifying the setting to be able to receive the intervention. Ideally, this judgment should be reached collectively. As such, the tool should be considered a **decision-support tool**, rather than an analysis or evaluation tool.

**Glossary**

**Intervention**: Generic term defining a community outreach program, a multi-strategy program, an intervention modality, etc.

**Primary intervention**: An intervention carried out for the first time in an experimental (research) or innovation context (carried out for the first time by providers using an evaluation system).

**Replica intervention**: An intervention that is the result of a transfer, i.e., the implementation of an intervention that was originally tried or implemented in another context.

**Applicability**: The extent to which the process of an intervention can be implemented in another setting.

**Transferability**: The extent to which the result of an intervention in a given context can be achieved in another setting.

Tool 1

Tool to support the design and description of an intervention with a view to making it potentially transferable

| **Population** |  |
| --- | --- |
| **IN DESIGNING MY INTERVENTION, I TAKE INTO ACCOUNT AND DESCRIBE:** |  |
| 1. **The epidemiologic and sociodemographic characteristics of the recipient population** | **□** |
| Subcriteria: Socioeconomic characteristics (rate of unemployment); demographic characteristics (age, sex); health status |  |
| 1. **The cognitive, cultural, social, and educational characteristics of the population** | **□** |
| Subcriteria: Cultural characteristics (lifestyle and representations of the world); cognitive characteristics (knowledge); socio-educational characteristics |  |
| 1. **The type of motivation in the intervention’s recipient population** | **□** |
| Subcriteria: Motivation derived from the intervention (financial incentive**,** interest, convivial atmosphere) and/or initial motivation (interest, enthusiasm, pleasure) |  |
| 1. **The accessibility of the intervention** | **□** |
| Subcriteria: Financial accessibility; geographic accessibility; sociocultural accessibility |  |
| 1. **The climate of trust between providers and recipients** | **□** |
| Subcriteria: Prior knowledge of each other; past experience of intervention; no interfering relationships (such as in hierarchical interactions) |  |
| 1. **The recipients’ perceptions of the intervention’s utility** | **□** |
| Subcriteria: The intervention is a priority; it is recognized as effective and as having merit |  |
| 1. **The demand coming from the population** | **□** |
| Subcriteria: Collecting and taking into account population demand |  |
| 1. **The population’s perceptions of their own health needs** | **□** |
| Subcriteria: Collecting and taking these perceptions into account in the intervention |  |
| 1. **The level of participation among participants** | **□** |
| Subcriterion: Rate of participation |  |
| 1. **The degree of involvement of recipients** | **□** |
| Subcriteria: Mobilization and involvement of recipients |  |
| **Environment** | |
| **IN DESIGNING MY INTERVENTION, I TAKE INTO ACCOUNT AND DESCRIBE:** |  |
| 1. **The institutional environment directly influencing the intervention** | **□** |
| Subcriteria: Pre-existing and durable political will; institutional support affirmed and announced by decision-makers; decision-makers’ positive perception of the intervention; equivalent status and latitude for action |  |
| 1. **Other elements of the implementation context** | **□** |
| Subcriteria: Prior synergistic experiences (such as raising awareness in the population) or antagonistic experiences; current existence of synergistic or antagonistic interventions (such as another intervention that is pursuing an objective that is in opposition to that of the replica intervention) |  |
| 1. **The partners enlisted for the intervention** | **□** |
| Subcriteria: Types of partners (e.g. community groups, associations, local decision-makers, funding organizations, public agencies); partners’ involvement |  |
| **IMPLEMENTATION** |  |
| **IN DESIGNING MY INTERVENTION, I TAKE INTO ACCOUNT AND DESCRIBE:** |  |
| 1. **The intervention methods** | **□** |
| Subcriteria: Strategic principles (educational activities, professional training, changes to the environment); action plan; implementation process; recipients’ involvement in planning (participative process); communication mechanisms in the intervention (e.g. between stakeholders); tools used; scale and duration of the intervention |  |
| 1. **The resources for the intervention** | **□** |
| Subcriteria: Financial and human resources |  |
| 1. **The capacities of the providers and the project leader** | **□** |
| Subcriteria: Competencies suited to the intervention (scientific / practical-experiential / know-how); appropriate expertise for the intervention, knowledge of the population’s characteristics, capacity for team work; competency regarding implementation of the primary intervention acquired through specific training |  |
| 1. **The mechanisms for motivating providers** | **□** |
| Subcriteria: Involvement; motivation; financial, scientific and/or professional interest |  |
| **SUPPORT FOR TRANSFER** |  |
| **IN DESIGNING MY INTERVENTION, I TAKE INTO ACCOUNT AND DESCRIBE:** |  |
| 1. **In communicating about my intervention, I include the elements that would be needed for transfer** | **□** |
| Subcriteria: based on normal means and structures (e.g. local professionals); evaluation of results and processes (elements that contributed to the results); availability of documents and tools used in the primary intervention (process, teaching tools, questionnaires) |  |

Tool 2

Tool to analyze the transferability of an intervention and to support transfer

| **Population** |  |
| --- | --- |
| 1. **The epidemiologic and sociodemographic characteristics of the recipient population are similar in the primary and replica interventions** | **□** |
| Subcriteria: Socioeconomic characteristics (rate of unemployment); demographic characteristics (age, sex); health status |  |
| 1. **The cognitive, cultural, social, and educational characteristics of the recipient population are similar in the primary and replica interventions** | **□** |
| Subcriteria: Cultural characteristics (lifestyle and representations of the world); cognitive characteristics (knowledge); socio-educational characteristics |  |
| 1. **The type of motivation in the recipient population is similar in the primary and replica interventions** | **□** |
| Subcriteria: Motivation derived from the intervention (financial incentive**,** interest, convivial atmosphere) and/or initial motivation (interest, enthusiasm, pleasure) |  |
| 1. **The intervention’s accessibility is similar in the primary and replica interventions** | **□** |
| Subcriteria: Financial accessibility; geographic accessibility; sociocultural accessibility |  |
| 1. **The climate of trust between providers and recipients is similar in the primary and replica interventions** | **□** |
| Subcriteria: Prior knowledge of each other; past experience of intervention; no interfering relationships (such as in hierarchical interactions) |  |
| 1. **The recipient population in the replica intervention believes in the utility of the primary intervention** | 1. **□** |
| Subcriteria: The intervention is a priority; it is recognized as effective and as having merit |  |
| 1. **The recipient population in the replica wants the intervention just as much as did the recipient population of the primary intervention** | **□** |
| Subcriteria: Collecting and taking into account population demand |  |
| 1. **The primary and replica populations have similar perceptions of their health needs** | **□** |
| Subcriteria: Collecting and taking these perceptions into account in the intervention |  |
| 1. **The intervention is equally acceptable to both the primary and recipient populations** | **□** |
| Subcriteria: Social, cultural, and philosophical acceptability (or conviction – e.g. if the intervention is a vaccination and the recipient is opposed to vaccination) |  |
| 1. **The levels of participation are the same in the primary and replica populations^[[3]](#footnote-3)^** | **□** |
| Subcriterion: Rate of participation |  |
| 1. **The recipients are involved to the same degree in both the primary and replica populations^[[4]](#footnote-4)^** | **□** |
| Subcriteria: Mobilization and involvement of recipients |  |
| **Environment** | |
| 1. **The institutional environment of the replica that has a direct influence on the intervention is supportive of its implementation and success** | **□** |
| Subcriteria: Pre-existing and durable political will; institutional support affirmed and announced by decision-makers; decision-makers’ positive perception of the intervention; equivalent status and latitude for action |  |
| 1. **Other elements of the replica implementation context are supportive of the intervention’s implementation and success** | **□** |
| Subcriteria: Prior synergistic experiences (such as raising awareness in the population) or antagonistic experiences; current existence of synergistic or antagonistic interventions (such as another intervention that is pursuing an objective that is in opposition to that of the replica intervention) |  |
| 1. **Partnerships in the primary and replica contexts are comparable** | **□** |
| Subcriteria: Same types of partners (e.g. community groups, associations, local decision-makers, funding organizations, public agencies); same level of partners’ involvement | |
| **IMPLEMENTATION** |  |
| 1. **The intervention methods in both the primary and replica interventions are similar** | **□** |
| Subcriteria: Strategic principles (educational activities, professional training, changes to the environment); action plan; implementation process; recipients’ involvement in planning (participative process); communication mechanisms in the intervention (e.g. between stakeholders); tools used; scale and duration of the intervention |  |
| 1. **The resources in the replica intervention are equivalent to those available in the primary intervention** | **□** |
| Subcriteria: Financial and human resources |  |
| 1. **The skills of the providers and the project leader in the replica intervention are similar to those in the primary intervention** | **□** |
| Subcriteria: Competencies suited to the intervention (scientific / practical-experiential / know-how); appropriate expertise for the intervention, knowledge of the population’s characteristics, capacity for team work; competency regarding implementation of the primary intervention acquired through specific training |  |
| 1. **The providers in the replica intervention believe in the utility of the primary intervention** | **□** |
| Subcriteria: Intervention recognized as a priority, effective, having merit; acceptance of the values and intervention methods upon which the intervention is based |  |
| 1. **The primary intervention is acceptable to the replica intervention’s providers** | **□** |
| Social, cultural, and philosophical acceptability (or conviction – e.g. if the intervention is a vaccination and the provider is opposed to vaccination) |  |
| 1. **The providers are mobilized in a similar manner in both the primary and replica interventions** | **□** |
| Subcriteria: Involvement; motivation; financial, scientific and/or professional interest |  |
| **SUPPORT FOR TRANSFER** |  |
| 1. **Adaptations can be (or were able to be) made to the primary intervention in the replica context without altering its fundamental nature** | **□** |
| Subcriteria: The context allows it; it is envisioned in the replica intervention’s process (diagnosis, programming that takes into account lessons from the primary intervention, project approach, management, continuous analysis of results to adjust the intervention) |  |
| 1. **The primary intervention has prepared and provided all the elements needed for its transfer** | **□** |
| Subcriteria: based on normal means and structures (e.g. local professionals); evaluation of results and processes (elements that contributed to the results); availability of documents and tools used in the primary intervention (process, teaching tools, questionnaires) |  |
| 1. **A knowledge transfer process exists in the replica setting** | **□** |
| Subcriteria: Support (scientific and methodological) provided to the intervention’s transfer; providers in the replica setting trained and supported in the primary intervention; links established with the promoter, providers, and/or evaluators of the primary intervention; lessons from the primary intervention taken into account |  |

1. AnalySe de la Transférabilité et accompagnement à l’Adaptation des Interventions en pRomotion de la santE [↑](#footnote-ref-1)
2. Wang S, Moss JR, Hiller JE. Applicability and transferability of interventions in evidence-based public health. Health Promot Int 2006 Mar;21(1):76-83.

   Cambon L, Minary L, Ridde V, Alla F. Transferability of interventions in health education: a review. BMC Public Health. 2012;12(1):497. [↑](#footnote-ref-2)
3. This question is intended for use in cases of *a posterior* evaluation, given that the data required will only be available at the end of the intervention. [↑](#footnote-ref-3)
4. This question is intended for use in cases of *a posterior* evaluation, given that the data required will only be available at the end of the intervention [↑](#footnote-ref-4)
